# A Case of Takotsubo Cardiomyopathy Triggered by Human Metapneumovirus

**DOI:** 10.7759/cureus.84977

**Published:** 2025-05-28

**Authors:** Brittani P Kongala, Mahesh Bhattarai, Hussam Ammar

**Affiliations:** 1 College of Medicine, Florida State University College of Medicine, Tallahassee, USA; 2 Internal Medicine, Tallahassee Memorial Hospital/Florida State University, Tallahassee, USA

**Keywords:** broken-heart syndrome, human metapneumovirus (hmpv), intensive care unit stay, reversible cardiomyopathy, severe sepsis, tako-tsubo cardiomyopathy

## Abstract

Takotsubo cardiomyopathy (TC) is a transient, left ventricular dysfunction and apical ballooning that is commonly associated with emotional or physical triggers; however, it is not a known complication of infection with human metapneumovirus (HMPV). This case report describes a 59-year-old woman with a history of anxiety, depression, seizure disorder, fibromyalgia, and chronic obstructive pulmonary disease, who initially presented to the emergency department with pneumonia and sepsis. A sputum polymerase chain reaction (PCR) was positive for HMPV. The following day after admission, the patient developed acute chest pain, increased troponin that peaked at 12.29 ng/L, and ST-segment elevation in L1, V2-V6 on ECG. Subsequent cardiac catheterization revealed normal coronary arteries, and an echocardiogram showed left ventricular systolic dysfunction with apical ballooning and an ejection fraction of 15%, consistent with a diagnosis of TC. To the best of our knowledge, this is the first reported case of TC in an adult secondary to infection with HMPV and thus marks an important contribution to the current knowledge of HMPV complications.

## Introduction

Takotsubo cardiomyopathy (TC), also known as stress-induced cardiomyopathy, transient apical ballooning, and broken heart syndrome, was first described in Japan in 1983 at Hiroshima City Hospital [1]. The first reported case was of a 64-year-old female patient who presented with acute chest pain and electrocardiographic features typical of acute myocardial infarction; however, the coronary arteries were normal, and there was an unusual left ventricular appearance that was characterized by a narrow neck and atypical ballooning during systole [2]. This characteristic appearance resembles “Takotsubo”, the Japanese word for “octopus pod” [1,3,4]. Remarkably, in the initial case, the significant wall-motion abnormalities resolved within two weeks on left ventriculography [2].

As seen in the initial case from Japan, TC is characterized by transient systolic and diastolic left ventricular dysfunction, accompanied by various wall-motion abnormalities with no coronary artery lesions to explain these changes. The current prevalence of TC is estimated to be 1-2% of patients with suspected acute coronary syndrome (ACS) and is an important differential diagnosis of ACS [1,3-6]. TC predominantly affects elderly women and is often preceded by an emotional or physical trigger, although the condition has been observed without an evident precipitating factor [1,5,6]. 

Contrary to its initial perception as a benign illness, TC has substantial mortality risks similar to those of ACS, such as life-threatening ventricular arrhythmia, cardiogenic shock, heart failure, and thromboembolism [1,3,6]. While TC can arise from sepsis, no adult cases of TC have been reported following infection with human metapneumovirus (HMPV) to the best of our knowledge. This case report documents the rare association of TC secondary to HMPV infection in a 59-year-old female patient.

## Case presentation

A 59-year-old female patient with a history of anxiety, depression, seizure disorder, fibromyalgia, and chronic obstructive pulmonary disease (COPD) presented to the emergency department (ED) with confusion. She was unable to answer questions, so her history was gathered from her husband, who brought her to the ED. The husband himself was recently hospitalized for pneumonia, and five days after his hospital discharge, his wife developed a productive cough and a fever. 

On admission, her vital signs were as follows: pulse 108/minute, respiratory rate 18/minute, blood pressure 95/73 mmHg, temperature 99.5°F, and oxygen saturation of 85% on room air. She had a Glasgow Coma Scale (GCS) of 14 (Best eye response 4, Best verbal response 4, best motor response 5). The physical examination revealed bilateral coarse breath sounds with bilateral expiratory wheezing and trace bilateral lower extremity edema. No focal neurological deficits were noted. The patient required bilevel positive airway pressure (BiPAP), and intravenous (IV) vancomycin and cefepime were started. The sputum polymerase chain reaction (PCR) was positive for HMPV, and chest CT showed bilateral ground-glass opacities.

**Figure 1:**
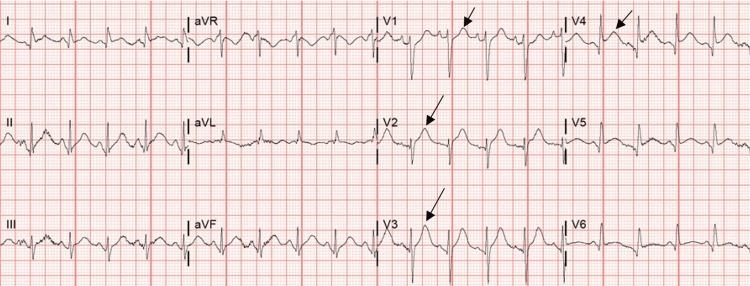
ECG showing anterolateral ST-segment elevation.

**Figure 2:**
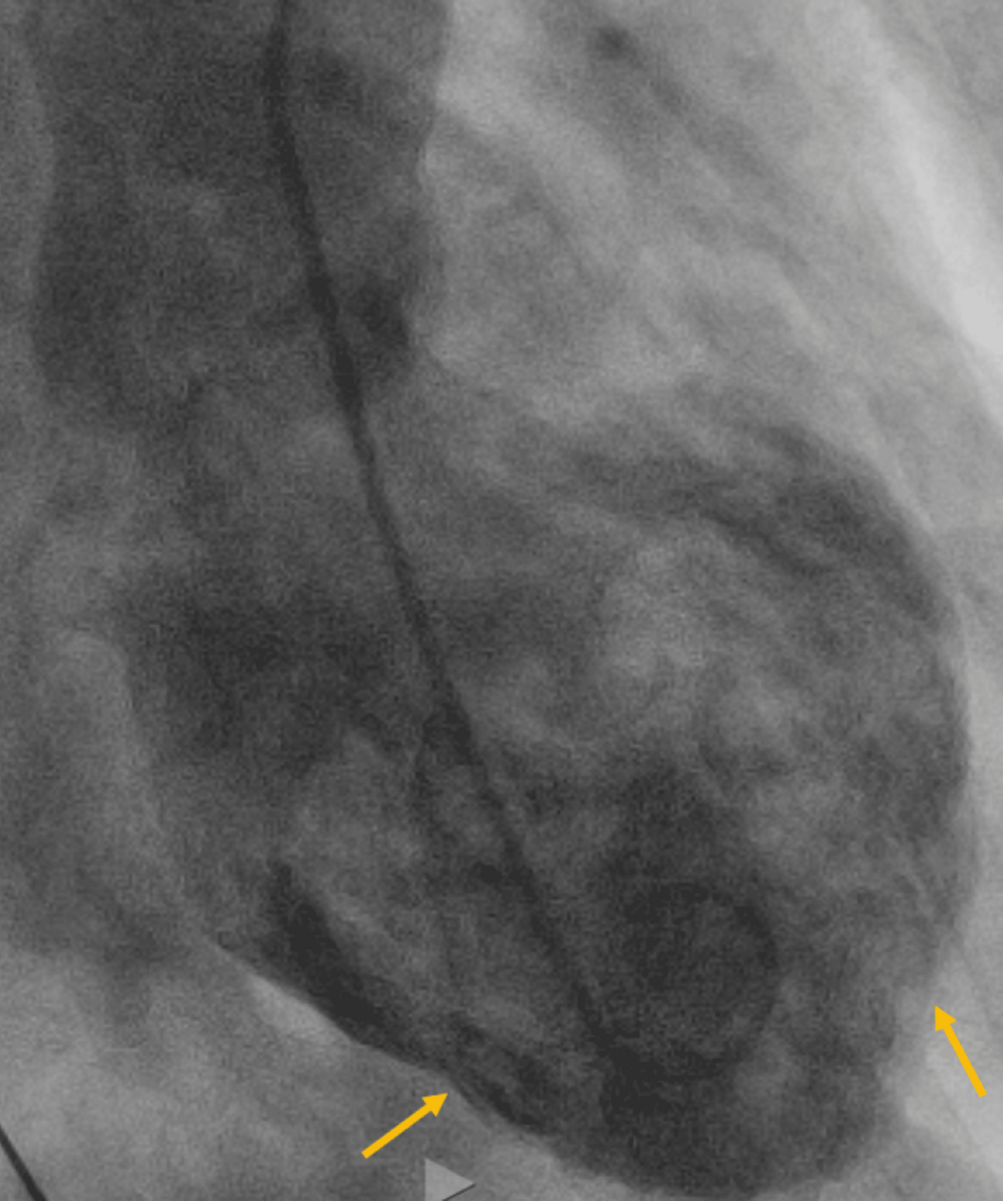
Cardiac catheterization and left ventricle ventriculogram revealing a hypokinetic apex.

**Figure 3:**
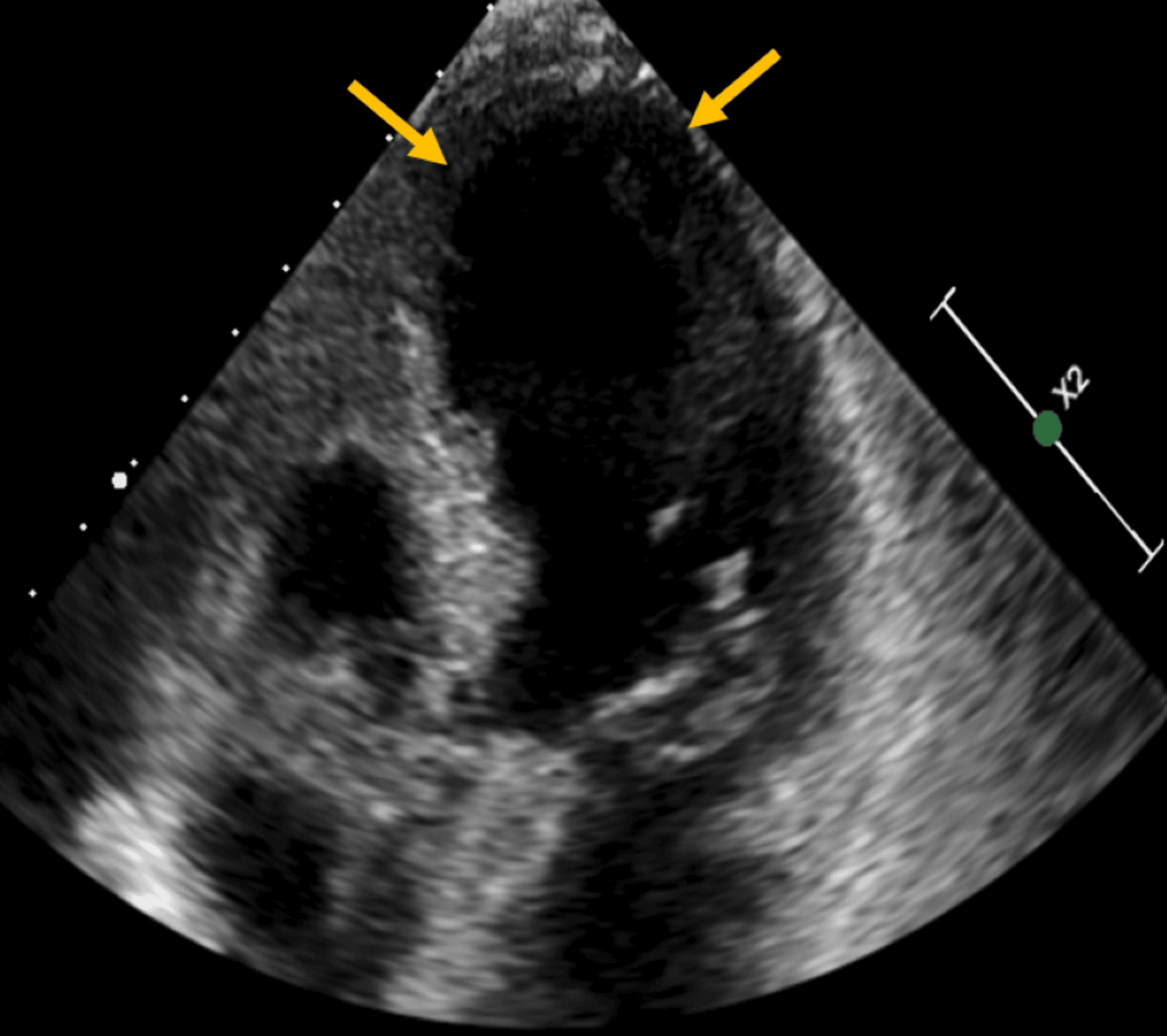
Echocardiogram on day 2 with hypokinetic apex and ejection fraction of 15%.

On the second day of her hospital admission, the patient experienced a decline in respiratory status, accompanied by chest pain, sinus tachycardia, and anterior ST-segment elevation in L1, V2-V6. Figure 1 shows the ECG. There was a dramatic increase in troponin from 0.29 ng/L to 12.29 ng/L. She was emergently taken to the cardiac catheterization suite for suspected ST-elevation myocardial infarction (STEMI). However, her coronary arteries were normal, but there was severe hypokinesis of the apex and inferior wall with an estimated ejection fraction (EF) of 15%, consistent with a diagnosis of TC. Figure 2 and Video 1 show her cardiac catheterization and the hypokinetic apex of the left ventricle. Figure 3 and Video 2 show her echocardiogram, which also confirms the hypokinetic apex of the left ventricle and supports the diagnosis of TC.

**Video 1:** Cardiac catherterization showing apical hypokinesia.

**Video 2:** Echocardiogram on day 2 of admission.

The patient developed several complications during her admission, including ventilator-associated pneumonia (VAP) on day 8. Sputum cultures identified *Burkholderia cepacia*, and she was treated with meropenem as indicated by the culture report. The patient also experienced status epilepticus with intermittent epileptiform discharges on electroencephalogram. This development was managed by initiating fosphenytoin and lacosamide, along with an increased dose of levetiracetam. 

**Figure 4:**
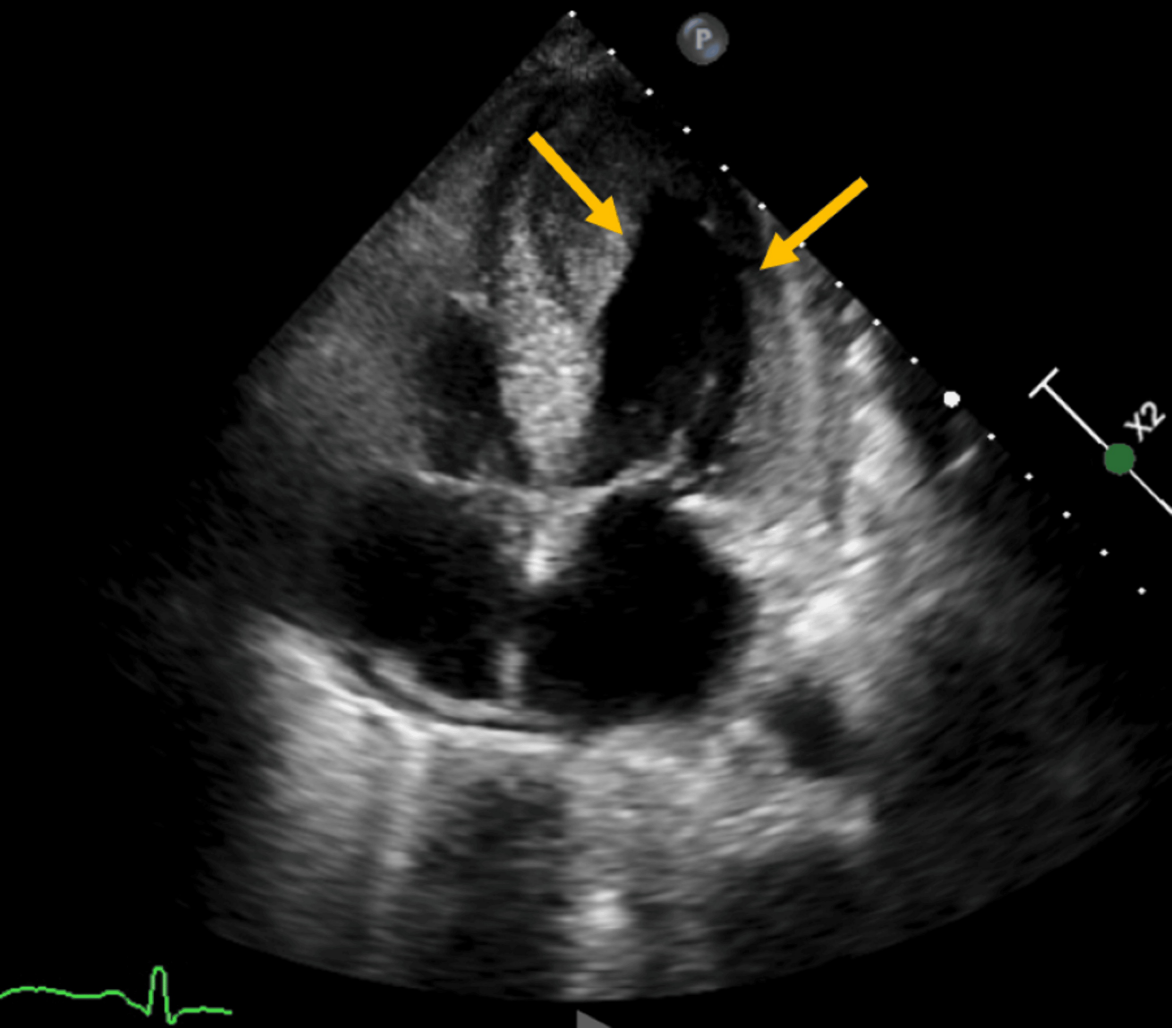
Echocardiogram on day 11 with normal ejection fraction.

A repeat echocardiogram on day 11 revealed normalization of her EF, as seen in Figure 4 and Video 3. However, a left ventricular (LV) thrombus had developed in the apex, which prompted the start of intravenous heparin infusion. While metoprolol and lisinopril were initiated upon the diagnosis of TC, they were held intermittently because of hypotension and the requirement of pressors. Furosemide was given as needed for volume overload and pulmonary congestion.

**Video 3:** Echocardiogram after recovery from Takotsubo cardiomyopathy.

On day 13, the patient was extubated and transitioned to noninvasive ventilation and high-flow oxygen. However, she was intubated again on day 16 because of respiratory distress and left lung collapse secondary to a mucous plug. In preparation for transition to a long-term acute care facility, she received a tracheostomy, and a Dobhoff tube was also inserted. A percutaneous gastrostomy tube could not be placed due to her prior history of gastric bypass surgery. Before discharge, the patient was transitioned from heparin to warfarin and bridged until reaching a therapeutic international normalized ratio (INR) of 2-3. On day 20, she was discharged to a long-term acute care facility, where she was eventually extubated. During her three-month follow-up, the patient returned to her baseline condition. She was fully independent in all her activities. Both her tracheostomy tube and percutaneous gastrostomy tube were removed, and an echocardiogram continued to reveal normal EF and resolution of the LV thrombus.

## Discussion

Despite being described over 30 years ago, the pathogenesis and pathophysiology of TC remain poorly understood [1-5]. Leading hypotheses suggest multiple potential mechanisms for the development of TC. These include catecholamine surge causing transient myocardial toxicity, aborted ST-elevation myocardial infarction with spontaneous thrombus lysis, coronary vasospasm, or microcirculatory dysfunction [3-5,7,8]. Although there is no existing literature on the potential mechanisms of TC secondary to HMPV, we speculate that the underlying mechanisms may be similar to those mentioned above.

Similar wall-motion abnormalities as seen in TC occur in other states of catecholamine excess, such as subarachnoid hemorrhage and pheochromocytoma [5]. However, TC has several unique characteristics that distinguish it from other acute cardiac emergencies. It is typified by transient LV dysfunction without coronary artery obstruction and unique anteroseptal-apical dyskinetic ballooning with hyperkinetic basal segments [5,8-10]. These abnormalities are transient and typically resolve spontaneously, restoring normal LV function and EF [8].

Risk factors for TC include emotional or physical stress and genetic factors [1,5]. In a cohort of 1,613 patients with TC, an emotional trigger was reported in one-third of patients, while physical triggers, including physical exertion, medical conditions, or procedures, were present in 39% [4]. Acute neurological disorders were identified in 6%, yet an estimated 31% of patients did not report an inciting event [4]. In addition, TC predominantly affects women (80-90% of cases) in the sixth decade of life, with patients under 50 years of age accounting for only 10% of cases [1,5]. In the case presented in the current report, the likely precipitant of TC was sepsis secondary to HMPV infection, and the patient’s age and sex are identified as risk factors that likely increased her susceptibility to TC. Her seizure disorder was well-controlled before her hospitalization and did not contribute to the development of TC.

As seen in this case, patients with TC classically present with acute-onset chest pain, dyspnea, and ECG changes, such as ST-segment elevation or depression and T-wave inversion [5,10]. Because this presentation mimics ACS, TC is often misdiagnosed and initially managed as ACS [1,5]. In extreme cases, TC can lead to severe heart failure, cardiogenic shock, or life-threatening arrhythmia requiring hemodynamic or ventilatory support [4]. Therefore, it is critical to differentiate TC from acute myocardial infarction. However, the diagnosis of TC usually only becomes evident during cardiac catheterization when no significant coronary artery disease is found on angiography [5,8-10]. Another differential diagnosis for TC is myocarditis. However, in the case of the current patient, the apical hypokinesis and rapid recovery of EF in less than two weeks are consistent with a diagnosis of TC and argue against the diagnosis of myocarditis. In this case, the cardiology team did not think there was a need for magnetic resonance imaging to make a diagnosis.

Sepsis is a known potential cause of TC, with cases reported from both gram-positive and gram-negative bacteremia [10,11]. *B. cepacia* is a gram-negative bacterium that is typically acquired in a healthcare setting, especially for patients on ventilators or those with a weakened immune system [12]. In the current case, *B. cepacia* was identified on the tracheal aspirate culture on day 8 of admission after the patient was suspected to have VAP. This suggests that the patient’s infection with *B. cepacia* was likely hospital-acquired and not the cause of her initial illness and subsequent development of TC.

Viral causes of TC, such as influenza and COVID-19, have also been reported as potential triggers [11,13]. However, HMPV infection is not a known cause of TC, and this makes the patient’s presentation unique. HMPV is a paramyxovirus that was discovered in 2001 and is associated with acute respiratory illness among infants and children across the globe [14]. HMPV also causes acute respiratory illness among older adults and people with underlying chronic conditions or immune deficiency, often resulting in hospitalization [14]. A small, retrospective review found that patients hospitalized with HMPV infection have an increased incidence of cardiac morbidity, such as atrial fibrillation with rapid ventricular rate, myocarditis, and ischemic events [15]. A PubMed search from 2000 to 2024 by the authors revealed no other cases of adult HMPV infection complicated by TC, highlighting the clinical significance of this rare association. This case adds to the current knowledge of HMPV complications and underscores the need for further research on HMPV-associated cardiomyopathies because of the potential clinical complications.

While only one other report of HMPV-associated TC in a pediatric patient [16] could be found, we could not find any reports of HMPV-associated TC in adults in a literature search. However, there have been a few reported cases of myocarditis related to HMPV infection in adults. One such case, which had similar features to our case, reported by Hennawi et al. was of a 58-year-old woman who presented with cardiogenic shock and was found to have an EF of 15-20% with apical ballooning and left ventricular thrombus [17]. Their patient tested positive for HMPV and quickly stabilized within two weeks to normal EF. Cardiac magnetic resonance imaging did not reveal any delayed myocardial enhancement. We believe that the absence of myocardial enhancement, the rapid recovery, and the apical ballooning seen on echocardiogram argue against a diagnosis of myocarditis and instead support a diagnosis of TC.

## Conclusions

Documented cases of HMPV-associated TC are scarce, and to the best of our knowledge, this case appears to be the first reported in adults, making it a unique and important contribution to the current knowledge of this rare association. HMPV should be recognized as a rare etiology of TC, and TC should be considered a potential complication of HMPV infection. Therefore, heightened clinical suspicion should remain for patients with HPMV infection who present with ACS symptoms and have risk factors for TC.
